# Solvent Switched Weak Interaction of a 4-Quinazolinone with a Cavitand Derivative

**DOI:** 10.3390/molecules25081915

**Published:** 2020-04-21

**Authors:** Zoltán Nagymihály, Beáta Lemli, László Kollár, Sándor Kunsági-Máté

**Affiliations:** 1 Department of Inorganic Chemistry, Faculty of Sciences, University of Pécs, Ifjúság 6, H 7624 Pécs, Hungary; nmzoltan@gamma.ttk.pte.hu (Z.N.); kollar@gamma.ttk.pte.hu (L.K.); 2 János Szentágothai Research Center, University of Pécs, Ifjúság 20, H-7624 Pécs, Hungary; beata.lemli@aok.pte.hu; 3 Institute of Organic and Medicinal Chemistry, Medical School, University of Pécs, Szigeti 12, H-7624 Pécs, Hungary; 4 Department of General and Physical Chemistry, Faculty of Sciences, University of Pécs, Ifjúság 6, H 7624 Pécs, Hungary

**Keywords:** cavitand, inclusion complex, thermodynamics, fluorescence

## Abstract

Interaction of 4-quinazolinone with tetrakis (3,5-dicarboxylatophenoxy)-cavitand derivative has been studied in methanol and dimethylformamide media using fluorescence spectroscopy and molecular modeling methods. Results show temperature dependent complex formation: either the entropy gain or the high enthalpy changes are responsible for the formation of stable complexes in two separated temperature regions. However, different thermodynamic parameters are associated to different conformations of the complexes: while the high entropy gain associated to the formation of deeply included guest in methanol, the high entropy gain is associated with the formation of weakly included guest in dimethylformamide solvent. This finding highlights the importance of dynamic properties of the species interacted in different solvents.

## 1. Introduction

Interactions of bioactive molecules with molecular capsules are the focus of research for several decades. This is because the molecular capsules either offer fine tuning of solubilization of molecules included or can be applied in selective sensing of such molecules. Cavitands are members of the calixarene family [1] possessing aromatic moieties [2], and in addition to the inorganic or organic ions [3,4] they are also able to form complexes with neutral molecules [5]. The delocalized electron cloud of the aromatics of the host cavitand derivative participates in both the photoluminescence (PL) process and the molecular interaction with the guest included, therefore, the change in the PL signal during the inclusion of the guest compound often reflects the amount of complex formed. Therefore, the very sensitive PL methods can be applied to determine the molecular interactions [6].

Further the complex formation inside the cavitand’s cavity, deepened cavitands, i.e., functional groups with a larger size located in the upper rim extend the application field. This plays as “upper cavity” of the molecule and possesses some flexibility, which allows potential guest molecules to enter and to be more or less surrounded by the walls of the host. In this way, the entrapment of guest molecules can be performed in two steps, either bound to the functional groups in the upper rim or deeply complexed by the aromatic building blocks of the cavitand skeleton. This series of deepened cavitands may be useful for embedding electron-poor aromatics. Considering the ability of binding specific chiral molecules and due to the presence of a flexible binding pocket with tunable electron-density of these hosts, applications in selective sensing of drugs are also the focus of this research.

However, in those cases when the host molecules offer different regions for binding the guest, the complexes formed can be associated with significantly different complex stability. In our previous work [7] the temperature dependent quenching of a cavitand derivative by copper ions has been investigated in dimethylformamide (DMF) media. Results identified two temperature regions where the temperature dependence of the interactions show a significant difference: accordingly, it is assumed that at lower temperatures the coordination of the Cu^2+^ ion takes place in the cavity while at higher temperature Cu^2+^ is coordinated to the outer part of the cavitand with its almost retained solvation shell. The complex stabilities are very similar in the two temperature ranges, however, their temperature dependencies are quite different. This property can be described by the enthalpy-entropy compensation: when the copper ions lose the solvation shell prior to entering into the cavitand cavity, the enthalpy gain associated to the formation of the cavitand-copper complex partly used to the desolvation of the copper ions. The energy lost is compensated by the entropy gain associated to the increased freedom of the DMF molecules. In contrast, when the copper ion does not enter into the cavity, the ions retain the solvation shell, therefore the missing entropy enhancement keeps the Gibbs free energy unchanged since the enthalpy change does not contain the enthalpy associated to the removal of the solvation shell.

However, the structure of the solvent (if any) plays an important role in the formation of the entropy gain: solvent molecules become to be free after the complex formation by leaving the solvation shell of the guest and leaving the cavity of the host. Afterwards, in cases of protic solvents, formation of clusters performed, which process decreases the entropy. No such entropy decreasing process exists in the cases of non-protic solvents, e.g., [7].

Following the synthesis and applications of a new cavitand derivative, in this work first steps towards understanding will be done, how the coordination of the guest molecule depends on the temperature and how this property is affected by the structure of the solvents. Accordingly, the complex formation of a drug molecule with a cavitand derivative has been compared in protic methanol and non-protic dimethylformamide solvents. Due to the possible applications in electrochemical sensors for in vivo measurements, 4-quinazolinone molecule known as the inhibitor of the DNA repair enzyme poly (ADP-ribose) polymerase (PARP) is chosen as the guest for these studies.

## 2. Results and Discussion

**Figure 1:**
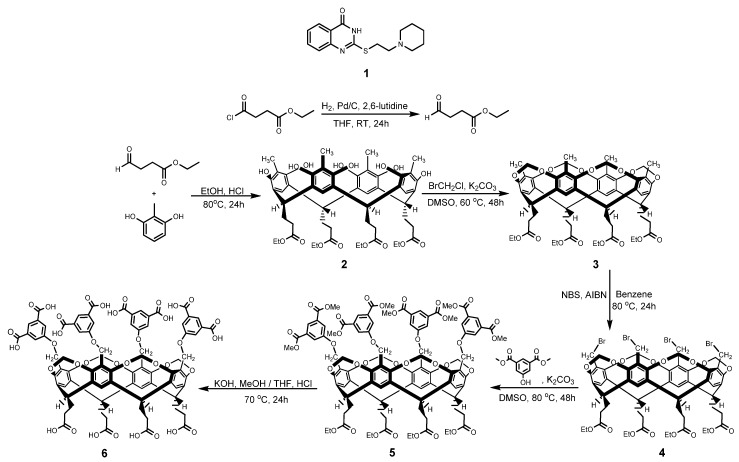
Interaction of 4-quinazolinone (**1**) with tetrakis(3,5-dicarboxylatophenoxy)-cavitand (**6**) was studied in dimethylformamide and methanol media. Synthesis of the host compound (**6**).

Tetrakis(3,5-dicarboxylatophenoxy)-cavitand, a highly polar cavitand derivative (**6**) possessing 12 carboxylate functionalities (8 on the upper rim, 4 on the lower rim) was synthesized in order to study host–guest interactions with compounds of pharmacological interest such as 4-quinazolinone (**1**) (Figure 1).

The parent cavitand **2** was obtained in the ring closure reaction of 2-methylresorcinol and ethyl 4-oxobutanoate, which can be derived from the commercially available acid chloride derivative via Rosenmund reaction. The upper rim of the ‘basket’ was closed by bromochloromethane resulting in **3**. Bromination in the benzylic positions was carried out using N-bromosuccinimide (NBS). The tetrabromocavitand (**4**) underwent a substitution reaction using dimethyl 5-hydroxyisophthalate as the nucleophile. The hydrolysis of the ester functionalities of **5** resulted in the formation of the target upper rim octacarboxylate (**6**) (Figure 1).

**Figure 2:**
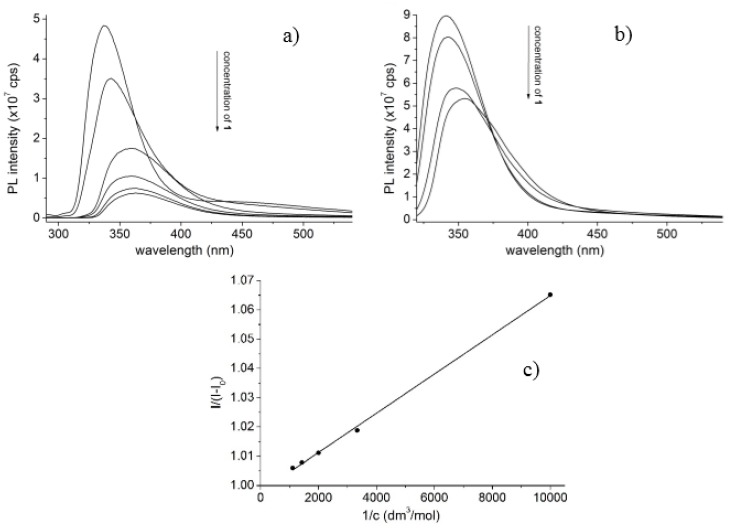
Emission spectra of **6** (100 μM) in the absence and in the presence with increasing concentration of **1** (0–900 μM) in (**a**) MeOH (λ_exc_ = 280 nm) or (**b**) DMF (λ_exc_ = 310 nm) solvents. (**c**) Representative Benesi–Hildebrand plot of the PL data to determine the stability constants (10^−4^ M of **6** upon increasing the concentration of **1** from 1.0 × 10^−4^ to 9.0 × 10^−4^ M).

Fluorescence spectra of the tetrakis(3,5-dicarboxylatophenoxy)-cavitand show decreased emission of **6** upon increased concentration of the guest **1**. Changes induced in the PL spectra of **6** in the presence of **1** highlight the interaction between the molecules, which the signal offers determination of the stability constants using the Benesi–Hildebrand method (Figure 2b). Accordingly, the stability constants were determined in the temperature range from 289.16 to 305.16 K with the step of 2 K. Three parallel measurements were performed and the average values of the stability constants were then used to calculate the thermodynamic parameters. Data evaluation also performed by the Hyperquad 2006 code. Comparison of the log K values derived by the two evaluation methods enclosed as Supplementary Materials.

**Figure 3:**
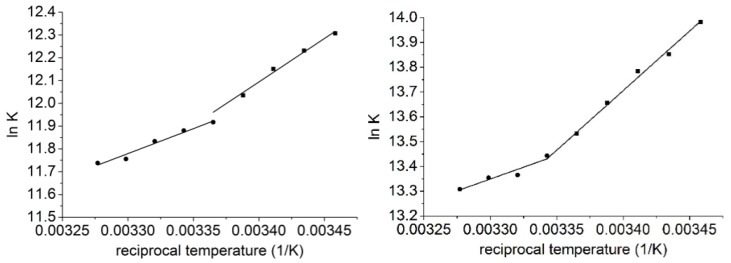
van’t Hoff plot to determine the thermodynamic parameters associated to the formation of **1**–**6** complexes in methanol (left) and in dimethylformamide (right) solvents.

Figure 3 shows the van’t Hoff plot of the species interacted, Table 1 summarizes the complex stabilities. The figure shows that two different temperature region could be identified where the thermodynamic parameters of the complex formation differed significantly (Table 2): in both solvents the complex formation of **1** with **6** was associated with negative entropy changes in the lower temperature region (289.16–297.16 K, methanol; 289.16–299.16 K, dimethylformamide), while positive entropy gains were associated in the higher temperature regions (297.16–305.16 K, methanol; 299.16–305.16 K, dimethylformamide). The significantly different thermodynamic parameters reflected different complex formation mechanisms in the different temperature regions. It is worth mentioning here, that the entropy gain in the higher temperature region was associated with weaker enthalpy gain. This behavior suggests the presence of enthalpy–entropy compensation: to remove the solvent molecules from the cavity costs energy, which weakens the enthalpy change while enhancing the entropy gain. Accordingly, on first view of the experimental results, it could be concluded that in the lower temperature range the guest molecules entered only into the upper rim of the host cavitand derivative and coordinates within the dicarboxylatophenoxy moieties. Since the bond between the dicarboxylatophenoxy moieties and the guest molecule inhibited the free motion of the dicarboxylatophenoxy arms, the entropy decreased to a high extent. Then, in the higher temperature regions the guest molecule **1** entered into the cavity of the cavitand skeleton. In these cases the solvent molecules have to leave the cavity prior complex formation, which processes the costs energy and also enhances the entropy term.

However, considering the solvent structures, they reflected discrepancies in the experimental results: this is because the formation of clusters by solvent molecules, which leave the solvation shells of the species interacted and also leave the cavity of the hosts, decreased the entropy gain. This observation can be clearly seen in the higher temperature region when the entropy gain obtained in the dimethylformamide solvent was higher than that of measured in methanol solutions. In contrast, in the lower temperature region lower entropy change was associated to the complex formation performed in dimethylformamide than in methanol media. This observation was in contrast with the description given above.

To get deeper insights at molecular level into the formation mechanism of the complexes in different media, molecular modeling studies were performed. The explicit solvation model combined with semiempirical molecular modeling was applied. The entropy term was calculated using the molecular vibrations determined in harmonic approximation.

**Figure 4:**
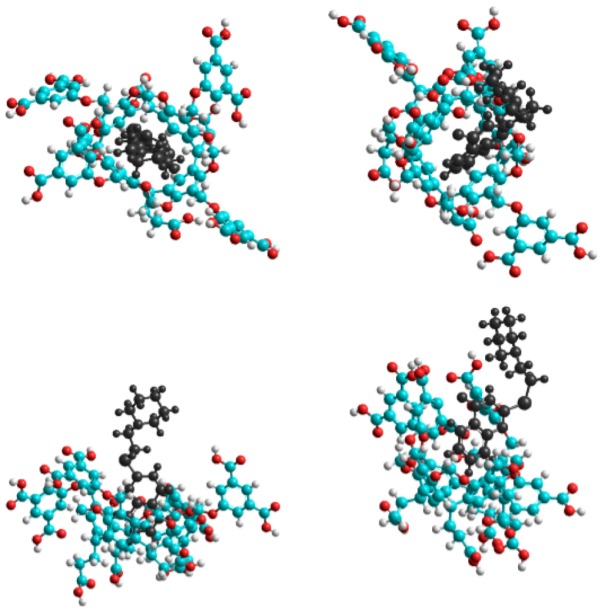
Top (**top**) and side (**bottom**) views of the equilibrium conformations of host–guest complexes of **1** with **6**. The guest **1** molecule enters into the cavitand cavity by its piperidine (**left**) or quinazoline moiety (**right**).

The complex formation without the solvation model was examined first. Table 3 summarizes the results and Figure 4 shows the equilibrium conformation associated to the complex formation. The conformation analysis resulted in two stable conformations at room temperature due to the fact that the guest **1** molecule can enter into the cavitand cavity either by its piperidine or quinazoline moiety. However, only the latter structure, which is based on the π–π interactions, was stable at room temperature. Although the C–H…π bonds between the C–H groups of the piperidine and the π-cloud of the cavitands phenyl could stabilize the former structure, the decreased entropy at room temperature inhibited the complex formation. Although the theoretical data are far from the experimental results, the enthalpy–entropy compensation can be clearly observed. It means that the reduced freedom of motion of the dimethylformamide moieties themselves can describe the quite different entropy change associated to the formation of complexes with two different conformations. It suggests that the role of the solvent molecules in the determination of the entropy was much weaker than assumed from the experimental results.

Considering explicitly the solvent molecules by the TIP3P model quite a different description rose to explain the complex formation. The results obtained for methanol solutions correlated with the experimental findings and the theoretical results derived in vacuum: the two stable conformations based either on the bonding between the quinazoline moiety of the guest and the dicarboxylatophenoxy moiety of the host, or when the guest entered deeper into the cavity of the host, the complex was stabilized by the interaction between the quinazoline moiety of the guest and the aromatic rings of the host skeleton. Results show very clearly (Table 4) that in the dimethylformamide media the thermodynamic parameters associated to the formation of the A and B structure shows the opposite relationship with the obtained data in methanol solutions. It has to be noted here that the theoretical data show very good agreement with the experiments.

**Figure 5:**
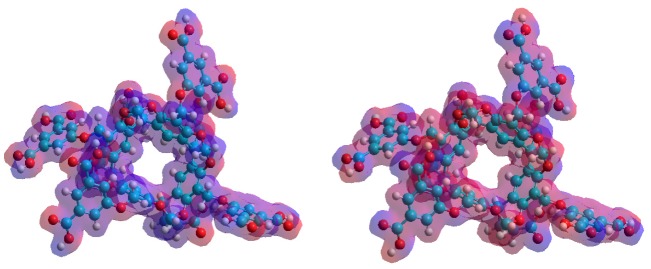
Kinetic energy distribution (Debye temperature) of host calixarene molecules dissolved in methanol (**left**) and dimethylformamide (**right**) solutions. Red color indicates higher while blue color indicates lower temperature regions.

In our previous studies we found several cases when the kinetic energy shows inhomogeneous and temperature-dependent distribution on the molecules, which is raised by the solute–solvent interactions [8,9]. The consequence of this redistribution, being in accordance with the Rice–Ramsperger–Kassel–Marcus (RRKM) theory [10], is that it could make force on the reaction pathways for the formation of host–guest complexes. Accordingly, we calculated the Debye temperature of the atoms in the host molecule dissolved in methanol or dimethylformamide solvents. Figure 5 shows the distribution of the kinetic energies on the cavitand host molecule in methanol or dimethylformamide solvent at 298 K. Results show enhanced atomic motions on the dicarboxylatophenoxy arms in methanol solutions while enhanced atomic motions on the cavitand core aromatic rings could be observed in dimethylformamide solutions.

Considering the RRKM theory, the enhanced motion of the reactants’ atoms weakened the bonds, therefore, on the contrary, distribution of the kinetic energy in the different solvent exchange the thermodynamic parameters associated to the complex formation of **1** guest with **6** host in methanol or dimethylformamide media.

## 3. Materials and Methods 

### 3.1. Chemicals

The 4-quinazolinone derivative (PARPI, 1) was synthesized as described earlier [11]. The methanol (HPLC grade) and anhydrous DMF (99.8% pure) were obtained from Sigma–Aldrich (Darmstadt, Germany).

### 3.2. Synthesis of Tetrakis(3,5-Dicarboxylatophenoxy)-Cavitand

The synthesis of cavitand **6** was carried out in the following steps.

#### 3.2.1. Synthesis of Ethyl 4-Oxobutanoate

Of 10% Pd/C 2.9 g was flushed with hydrogen, anhydrous THF was added and the solution was reflushed with hydrogen. 2,6-Lutidine (21.2 g, 198 mmol) and ethyl 4-chloro-4-oxobutanoate (29.8 g, 181 mmol) were added, and the solution was stirred at room temperature under atmospheric hydrogen for 24 h. The reaction mixture was filtered through Celite and evaporated. The crude residue was dissolved again in CH_2_Cl_2_ (500 mL) and washed with water (200 mL), 1 M hydrochloric acid (200 mL) and again with water. The organic layers were dried on Na_2_SO_4_ and evaporated to dryness. The resulting residue was purified by silica gel column chromatography (hexane:ethyl acetate = 4:1) to obtain ethyl 4-oxobutanoate. Yield: 20.1 g/85%. ^1^H NMR (500.1 MHz, CDCl_3_): δ 1.22 (t, *J* = 7.1 Hz, 3H), 2.58 (t, *J* = 6.6 Hz, 2H), 2.75 (t, *J* = 6.6 Hz, 2H), 4.07 (q, *J* = 7.1 Hz, 2H), 9.74 (s, 1H). ^13^C {^1^H} NMR (125 MHz, CDCl_3_): δ 14.3, 26.8, 38.7, 61.0, 172.5, 200.4.

#### 3.2.2. Synthesis of Cavitand **2**

2-Methyl-resorcinol (3.97 g, 32.00 mmol) was dissolved in a mixture of 50 mL of ethanol and 10 mL of 37% hydrochloric acid in an ice-water bath cooled 100 mL round bottom flask. Ethyl 4-oxobutanoate (4.37 g, 33.60 mmol) was added dropwise to the reaction mixture. The reaction mixture was stirred at ice-water bath temperature for 30 min, then the flask was refluxed at 80 °C overnight. The precipitate formed was poured into precooled 200 mL of distilled water, filtered and dried under vacuum at 80 °C. Yield: 5.29 g/70%. ^1^H NMR (500.1 MHz, DMSO-*d*_6_): δ 1.18 (t, *J* = 7.1 Hz, 12H), 1.95 (s, 12H, Ar–CH_3_), 2.18 (t, *J* = 6.6 Hz, 8H), 2.51 (t, *J* = 6.6 Hz, 8H), 4.04 (q, *J* = 7.1 Hz, 8H), 4.25 (q, 4H, –CH_2_-CH), 7.36 (s, 4H, Ar–H), 8.69 (s, 8H, OH). ^13^C {^1^H} NMR (125 MHz, DMSO-*d*_6_): δ 10.4, 14.5, 28.7, 32.9, 34.5, 60.1, 112.3, 121.6, 124.6, 149.8, 173.3.

#### 3.2.3. Synthesis of Cavitand **3**

Cavitand **2** (1.89 g, 2.0 mmol) and K_2_CO_3_ (1.38 g, 10 mmol) were dissolved in 50 mL of DMSO in a 100 mL round-bottom flask under argon flowing. The mixture was equipped with a magnetic stirrer and stirred for one hour at room temperature. Subsequently, ClCH_2_Br (1.55 g, 12.0 mmol) was added to the reaction mixture, the flask was stirred at 60 °C for 48 h under argon atmosphere. The mixture was cooled to room temperature and poured into 250 mL of 2% hydrochloric acid. The precipitate was filtered through a glass filter and washed with ice cold water and dried under vacuum at 80 °C. Yield: 1.83 g/92%. ^1^H NMR (500.1 MHz, CDCl_3_): δ 1.29 (t, *J* = 7.1 Hz, 12H), 1.96 (s, 12H, Ar–CH_3_), 2.37 (t, *J* = 6.6 Hz, 8H), 2.58 (tt, *J* = 6.6 Hz, 8H), 4.17 (q, *J* = 7.1 Hz, 8H), 4.26 (d, *J* = 7.6 Hz, 4H, inner of OCH_2_O), 4.84 (q, 4H, –CH_2_–CH), 5.89 (d, *J* = 7.6 Hz, 4H, outer of OCH_2_O), 7.02 (s, 4H, Ar–H). ^13^C {^1^H} NMR (125 MHz, CDCl_3_): δ 10.3, 14.2, 25.3, 32.8, 36.6, 60.4, 98.4, 117.1, 124.1, 137.3, 153.6, 173.1.

#### 3.2.4. Synthesis of Cavitand **4**

Cavitand **3** (1.99 g, 2.0 mmol), NBS (2.85 g, 16 mmol) and AIBN (0.263 g, 0.16 mmol) were dissolved in 50 mL of benzene in a 100 mL round-bottom flask under argon. The reaction mixture was stirred at 80 °C for 24 h under argon atmosphere. The reaction mixture was filtered through celite and evaporated. The crude residue was dissolved again in CH_2_Cl_2_ and washed with water. The organic layers were dried and evaporated to dryness. The resulting residue was washed with 10 mL methanol, the precipitate was filtered through a glass filter and washed with small portions of methanol and dried under vacuum at 80 °C. Yield: 2.2 g/84%. ^1^H NMR (500.1 MHz, CDCl_3_): δ 1.29 (t, *J* = 7.1 Hz, 12H), 2.36 (t, *J* = 6.6 Hz, 8H), 2.60 (t, *J* = 6.6 Hz, 8H), 4.18 (q, *J* = 7.1 Hz, 8H), 4.43 (s, 8H, Ar–CH_2_–), 4.60 (d, *J* = 7.6 Hz, 4H, inner of OCH_2_O), 4.86 (q, 4H, –CH_2_–CH), 6.05 (d, *J* = 7.6 Hz, 4H, outer of OCH_2_O), 7.22 (s, 4H, Ar–H). ^13^C {^1^H} NMR (125 MHz, CDCl_3_): δ 14.2, 25.3, 32.5, 36.5, 60.6, 99.2, 120.1.1, 125.1, 126.4, 137.4, 153.8, 173.1.

#### 3.2.5. Synthesis of Cavitand **5**

Dimethyl 5-hydroxyisophthalate (2.10 g, 10 mmol) and K_2_CO_3_ (2.07 g, 15 mmol) were dissolved in 50 mL of DMSO in a 100 mL round-bottom flask under argon. The mixture was equipped with a magnetic stirrer and stirred for one hour at room temperature. Subsequently, Cavitand **4** (1.31 g, 1.0 mmol) was added to the reaction mixture, the flask was stirred at 80 °C for 48 h under argon atmosphere. The mixture was cooled to room temperature and poured into 250 mL of 2% hydrochloric acid. The precipitate was filtered through a glass filter and washed with ice cold water and small portion of n-hexane, and dried under vacuum at 80 °C. Yield: 1.64g/90%. ^1^H NMR (500.1 MHz, CDCl_3_): δ 1.31 (t, *J* = 7.1 Hz, 12H), 2.43 (t, *J* = 6.6 Hz, 8H), 2.69 (t, *J* = 6.6 Hz, 8H), 3.86 (s, COOMe, 24H), 4.21 (q, *J* = 7.1 Hz, 8H), 4.62 (d, *J* = 7.6 Hz, 4H, inner of OCH_2_O), 4.96 (q, 4H, –CH_2_–CH), 5.00 (s, 8H, Ar–CH_2_–), 5.83 (d, *J* = 7.6 Hz, 4H, outer of OCH_2_O), 7.36 (s, 4H, Ar–H). 7.78 (s, 8H, Ar–H), 8.26 (s, 4H, Ar–H). ^13^C {^1^H} NMR (125 MHz, CDCl_3_): δ 14.2, 25.3, 32.5, 36.5, 52.3, 61.4, 99.9, 120.0, 120.8, 120.9, 122.7, 125.1, 126.4, 137.4, 139.1, 153.8, 165.9, 173.1.

#### 3.2.6. Synthesis of Cavitand **6**

Cavitand **5** (0.91 g, 0.5 mmol) was dissolved in 20 mL of THF in a 100 mL round-bottom flask, then 3 cm^3^ Claisen’s alkali (prepared by dissolving 350 g of KOH in 250 cm^3^ of water, cooling and diluting to 1 L with MeOH) was added to the reaction mixture). The reaction mixture was refluxed at 70 °C for 24 h. The mixture was cooled to room temperature and the solution was acidified with 2 M hydrochloric acid. The precipitate was filtered through a glass filter and washed with ice cold water and small portion of n-hexane, and dried under vacuum at 80 °C. Yield: 528 mg/66%. ^1^H NMR (500.1 MHz, DMSO-*d*_6_): δ 2.32 (t, *J* = 6.6 Hz, 8H), 2.73 (tt, *J* = 6.6 Hz, 8H), 4.47 (d, *J* = 7.6 Hz, 4H, inner of OCH_2_O), 4.77 (q, 4H, –CH_2_–CH), 4.92 (s, 8H, Ar–CH_2_–), 5.88 (d, *J* = 7.6 Hz, 4H, outer of OCH_2_O), 7.66 (s, 8H, Ar–H), 7.84 (s, 4H, Ar–H). 8.07 (s, 4H, Ar–H), 10.30 (bs, 4H, COOH), 13.25 (bs, 8H, COOH). ^13^C {^1^H} NMR (125 MHz, DMSO-*d*_6_): δ 30.9, 33.1, 36.9, 61.4, 100.0, 119.8, 122.7, 123.0, 125.4, 133.1, 139.6, 153.7, 159.1, 166.8, 178.9.

### 3.3. Fluorescence Measurements

Fluorescence emission spectra were recorded on a Fluorolog τ3 Horiba Jobin Yvon spectrofluorimeter (Longjumeau, France) equipped with a Peltier thermostat (Jobin Yvon/SPEX, Longjumeau, France). The excitation wavelength was set to 295 nm. The fluorescence emission intensities within the scanning wavelength ranging from 310 to 500 nm were recorded by the right angle detection based optical arrangement. The stock solutions (2 × 10^−4^ M) of **1** PARPI and **6** cavitand was prepared in DMF and methanol solvent. During the measurements the concentration of the **6** host was kept constant at 1.0 × 10^−4^ M, while the concentration of **1** was varied between 1.0 × 10^−5^ and 1.0 × 10^−4^ M. The emission spectra were recorded at 11 different temperatures within the range from 289.16 to 305.16 K with 2 K steps. To determine the stability constants, data were corrected for the primary and secondary inner filter effects using the following approximation [12]:

(1) $$F_{corr}=F_{obs}*antilog\left(\frac{OD_{exc}+OD_{em}}{2}\right)$$

where *OD_exc_* and *OD_em_* are the optical densities associated to the excitation and emission wavelengths of the sample.

Binding constants (*K*, with the dimension of dm^3^/mol) of PARPI—cavitand complexes were calculated employing the graphical application of the Benesi–Hildebrand equation assuming 1:1 stoichiometry:

(2) $$\frac{I_{0}}{\left(I-I_{0}\right)}=\frac{1}{A}+\frac{1}{A*K*{\left[CD\right]}^{}}$$

where *I*_0_ and *I* denote the fluorescence emission intensities of **6** in the absence and in the presence of the guest, respectively and [*C*] is the molar concentration of the guest molecule while A is a constant.

### 3.4. Molecular Modeling

Thermodynamic parameters of the host–guest complexes were determined at 298 K as follows. The thermodynamic functions were calculated as the change of the energy or entropy by subtracting the total energies or entropies of the reactants from the total energies or entropies of the products. The entropy terms of the species interacted were calculated applying the Boltzmann-statistics. The higher contribution to the entropy comes from the vibrational motions. Therefore, after calculating the vibrational frequencies using the harmonic approximation, the entropy was then determined as the following equation implemented in the HyperChem code:

(3) $$S_{vib}=R\sum_{i}\{\frac{h\nu_{i}/kT}{e^{\left(h\nu_{i}/kT\right)}-1}-\ln[1-e^{\left(-h\nu_{i}/kT\right)}]\}$$

Here *ν_i_* is the frequency of vibration and *T* is the temperature (298.16 K).

The total energies of the species interacted have been calculated at the semiempirical AM1 level using the HyperChem 8 code. After the geometry optimization at the AM1 level the vibrational–rotational analysis was performed in harmonic approximation using the same AM1 approximation. Molecular environment was considered by the TIP3P solvation model implemented in HyperChem code [13] using an appropriate extension by Bender [14].

## 4. Conclusions

Experimental and theoretical studies were performed on the interaction of the 4-quinazolinone inhibitor with a cavitand derivative. Results show temperature dependent complex formation: in two separated temperature regions either the entropy gain or the high enthalpy changes were responsible for the formation of stable complexes. However, the thermodynamic parameters were associated with different conformations of the complexes: while the high entropy gain associated to the formation of deeply included guest in methanol solvent, the high entropy gain was associated to the formation of weakly included guest in dimethylformamide solvent. This finding highlights the importance of dynamic properties of the species interacted in different solvent.

## Figures and Tables

**Table 1:** Complex stability constants (log K values) associated to the complex formation of **1** with **6** in methanol or the DMF solvent determined for different temperatures.

Solvent	Temperature (K)								
	289.16	291.16	293.16	295.16	297.16	299.16	301.16	303.16	305.16
Methanol	5.34	5.31	5.27	5.22	5.17	5.15	5.13	5.1	5.09
DMF	6.07	6.01	5.98	5.93	5.87	5.83	5.8	5.79	5.77

**Table 2:** Thermodynamic parameters associated to the complex formation of **1** with **6** in methanol or the DMF solvent.

Temperature Range (K)	ΔH (kJ/mol)	ΔS (J/K*mol)
Methanol		
289.16–297.16	−34.16 ± 0.18	−15.88 ± 0.56
297.16–305.16	−17.23 ± 0.18	40.89 ± 0.26
Dimethylformamide		
289.16–299.16	−39.14 ± 0.18	−19.32 ± 0.36
299.16–305.16	−19.33 ± 0.18	46.93 ± 0.36

**Table 3:** Thermodynamic parameters associated with the complex formation of **1** with **6** in vacuum. Conformation A reflects binding of **1** guest to the dicarboxylatophenoxy moiety while conformation B reflects binding of the guest in the cavitand core cavity.

Conformation	ΔH (kJ/mol)	ΔS (J/K*mol)
A	−27.08	−21.88
B	−21.13	32.12

**Table 4:** Thermodynamic parameters associated to the complex formation of **1** with **6** in methanol or the DMF solvent simulated by the TIP3P solvent model.

Conformation	ΔH (kJ/mol)	ΔS (J/K*mol)
Methanol		
A	−35.26	−14.78
B	−19.13	39.19
Dimethylformamide		
B	−38.88	−18.22
A	−20.32	45.23

## Supplementary Materials

The following are available online, Table S1: Results derived by the Benesi-Hildebrand method, Table S2: Results derived by the Hyperquad code.
