# Supplementary material for: Solvent Switched Weak Interaction of a 4-Quinazolinone with a Cavitand Derivative
Source: Molecules. 2020 Apr 21;25(8):1915. doi: 10.3390/molecules25081915 (PMC7221616; doi:10.3390/molecules25081915)
Supplement: Supplementary file 1 [file molecules-25-01915-s001.pdf]

Solvent switched weak interaction of a 4-quinazolinone inhibitor with a cavitand derivative

Zoltán Nagymihály, Beáta Lemli, László Kollár and Sándor Kunsági-Máté

Supplementary Material

Content

^{1}H and ^{31}P NMR spectra of Cavitand **2-6** p2-p6
Complex stability constants (log K values) p7

^{1}H and ^{31}P NMR spectra of Cavitand **2**

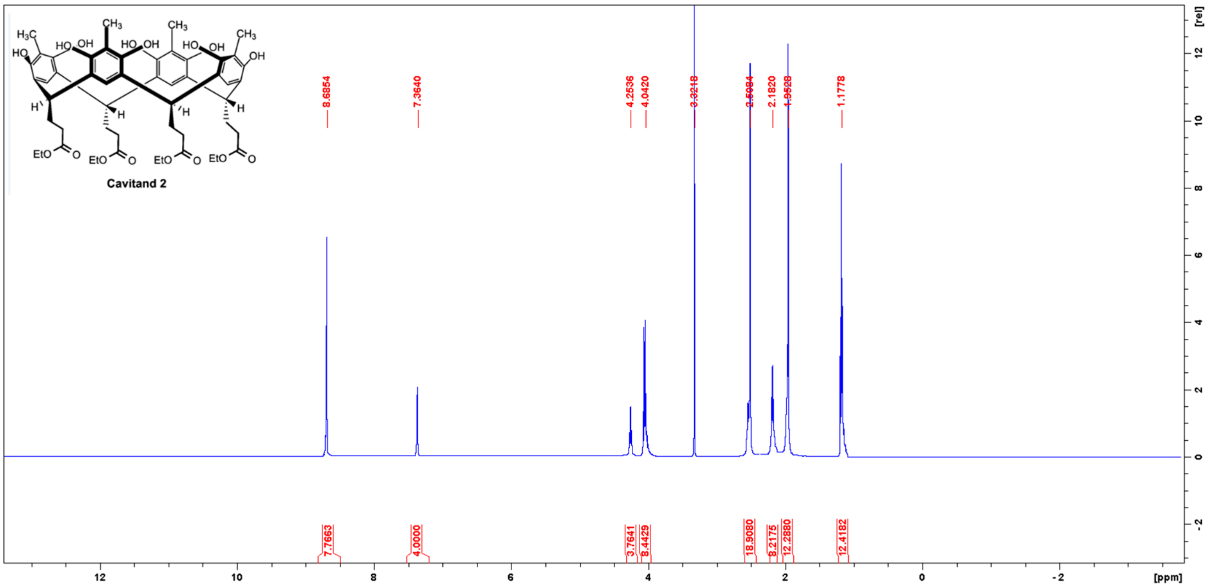

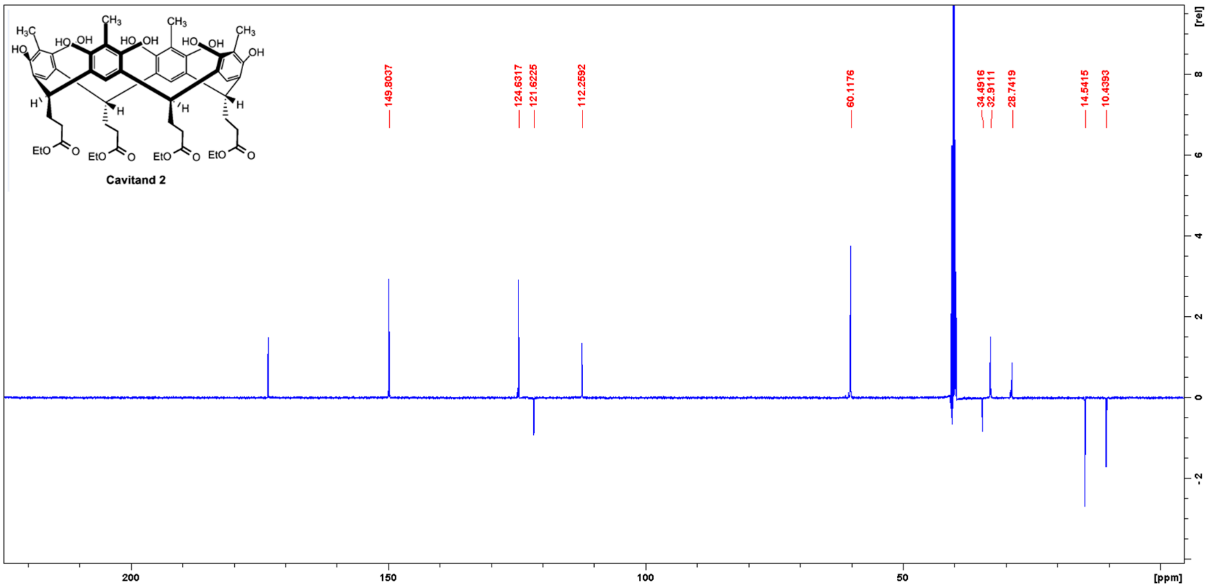

^{1}H and ^{31}P NMR spectra of Cavitand **3**

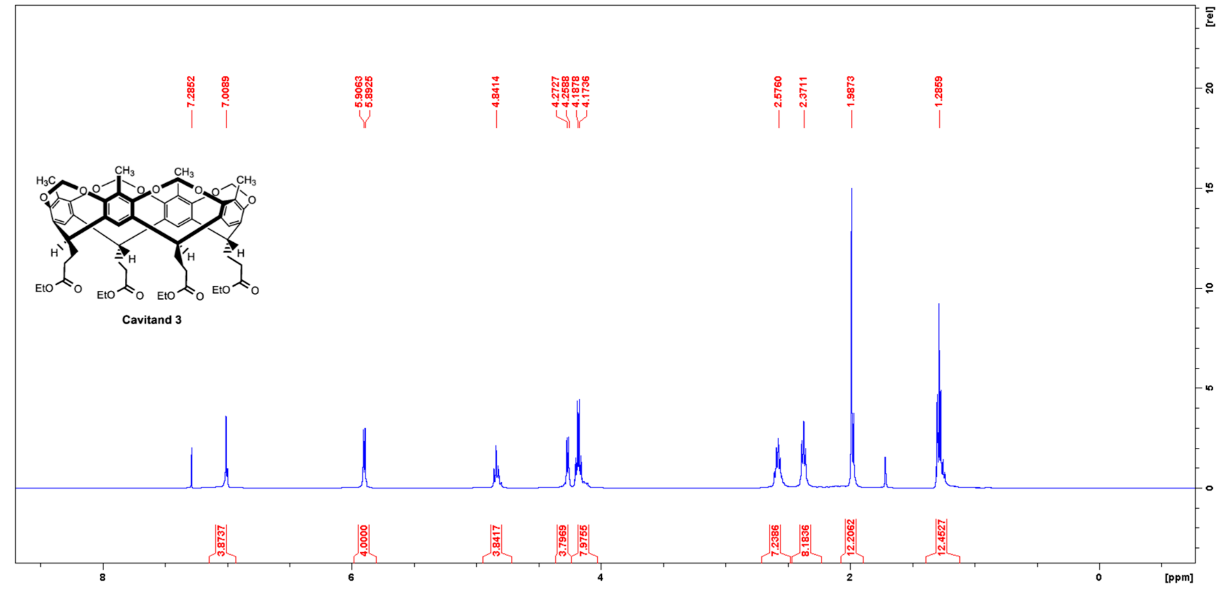

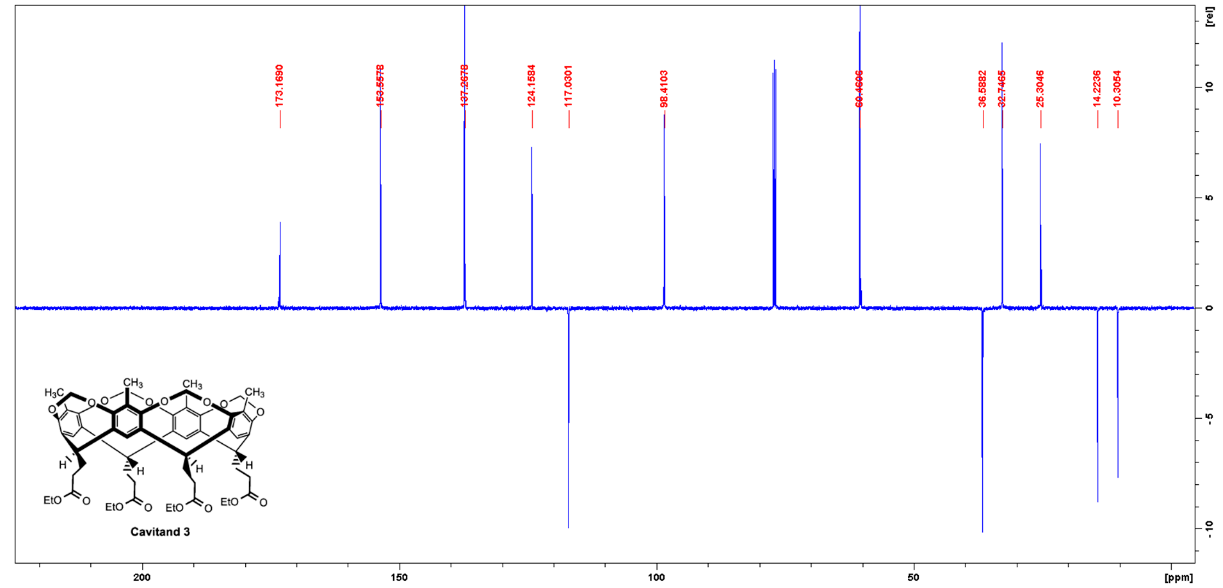

^{1}H and ^{31}P NMR spectra of Cavitand **4**

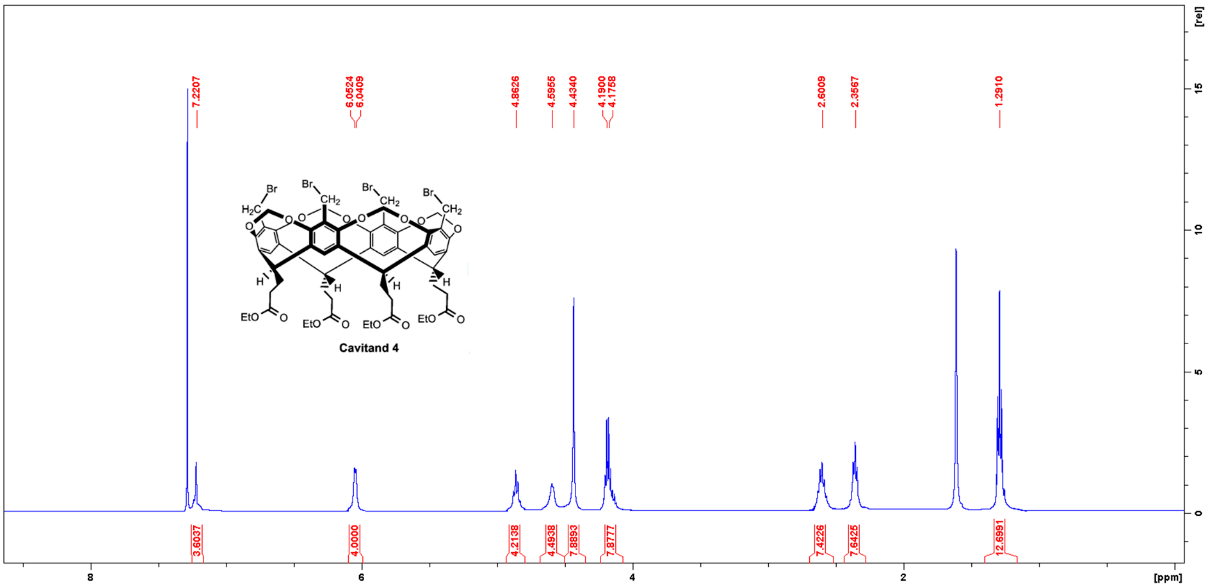

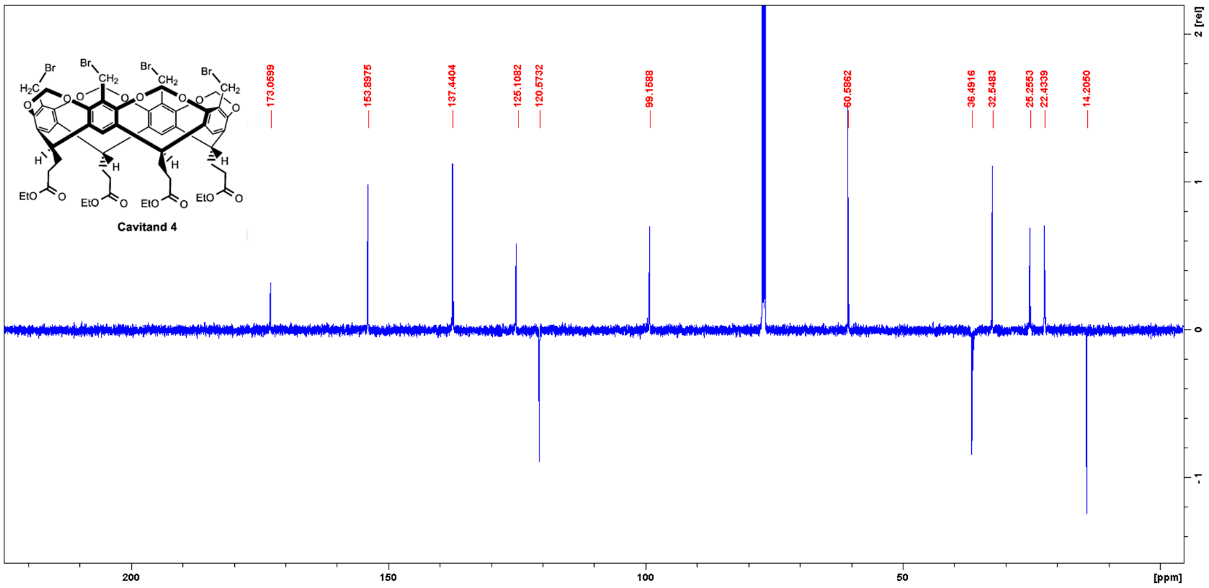

^{1}H and ^{31}P NMR spectra of Cavitand **5**

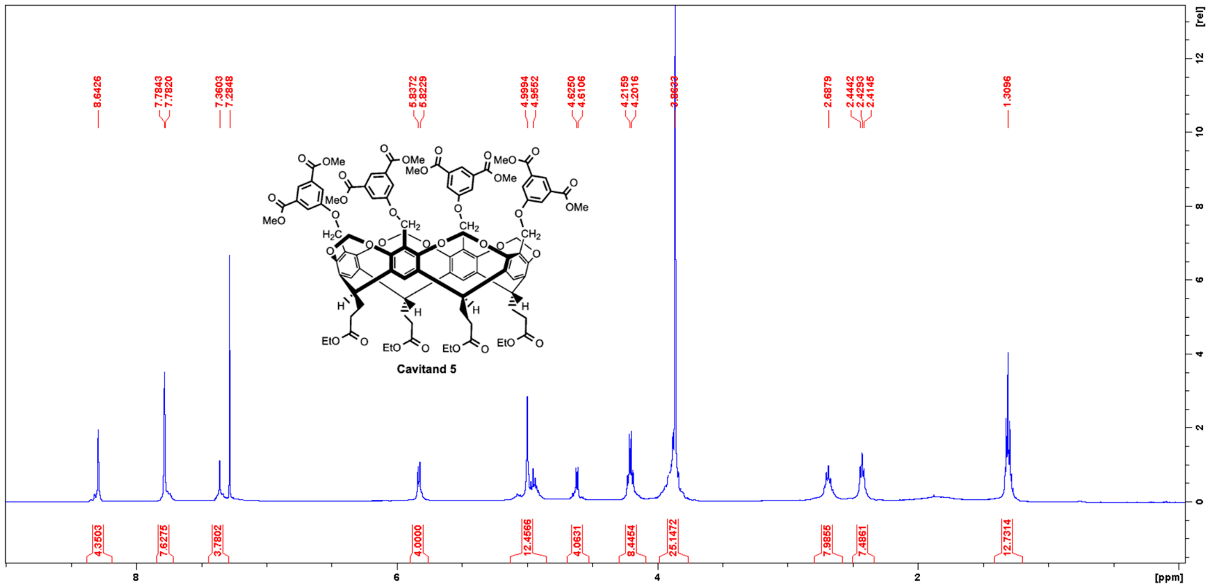

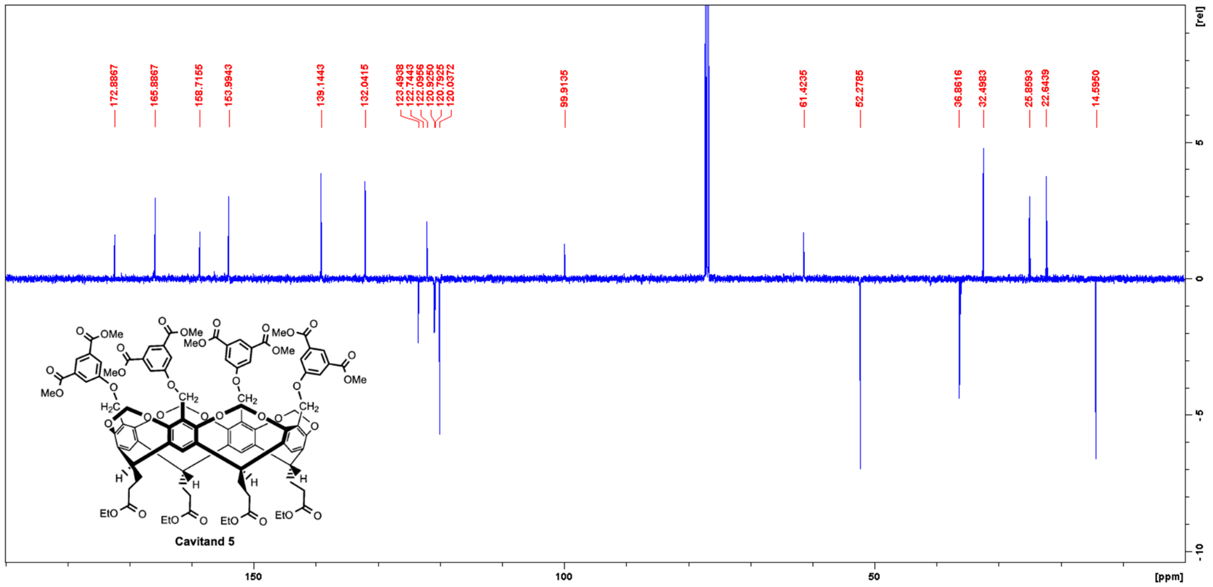

^{1}H and ^{31}P NMR spectra of Cavitand **6**

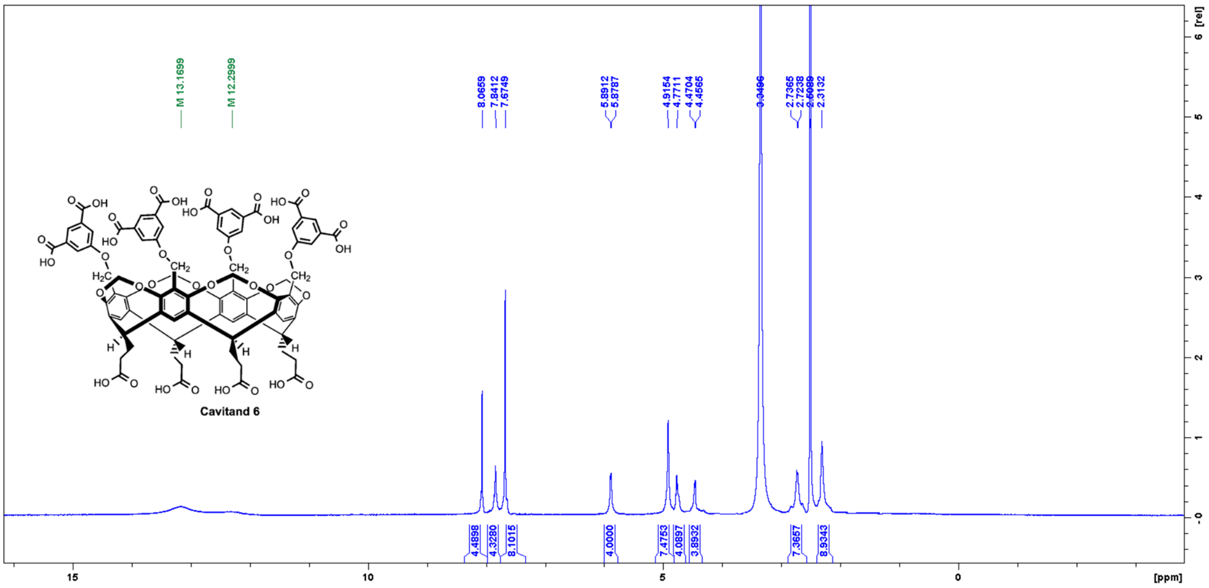
M 13.1699
M 12.2999
8.0659
7.8412
7.6749
5.8912
5.8787
4.9154
4.7711
4.4704
4.4565
3.3496
2.7365
2.7238
2.3132
4.4898
4.3280
8.1015
4.0000
7.4753
4.0897
3.8932
7.3657
8.9343
Cavitand 6
[ppm]
[rel]

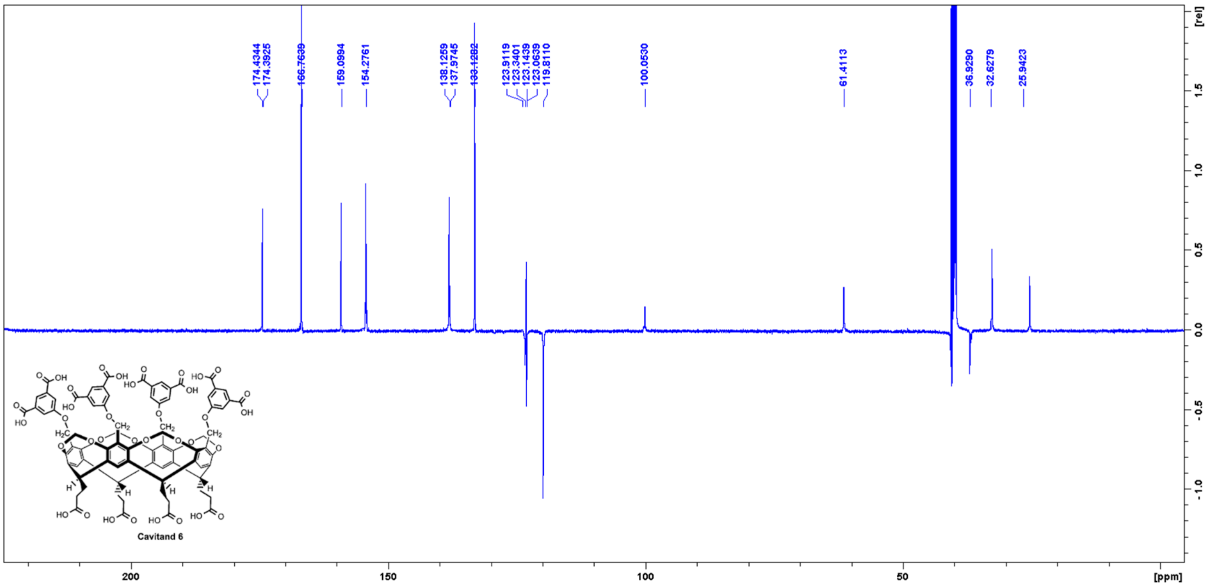
174.4344
174.3925
166.7639
159.0994
154.2761
138.1259
137.9745
133.1282
123.9119
123.3401
123.1439
123.0639
119.8110
100.0530
61.4113
36.9290
32.6279
25.9423
Cavitand 6
[ppm]
[rel]

Complex stability constants (log K values) associated to the complex formation of **1** with **6** in methanol or DMF solvent determined in the different temperatures.

Table S1: results derived by the Benesi-Hildebrand method

solvent	Temperature (K)								
	289.16	291.16	293.16	295.16	297.16	299.16	301.16	303.16	305.16
methanol	5.34	5.31	5.27	5.22	5.17	5.15	5.13	5.1	5.09
DMF	6.07	6.01	5.98	5.93	5.87	5.83	5.8	5.79	5.77

Table S2: results derived by the Hyperquad code

solvent	Temperature (K)								
	289.16	291.16	293.16	295.16	297.16	299.16	301.16	303.16	305.16
methanol	5.32	5.30	5.25	5.22	5.16	5.15	5.13	5.11	5.09
DMF	6.06	6.03	5.99	5.94	5.86	5.83	5.82	5.80	5.78
